# Quality of opioid prescribing in older adults with or without Alzheimer disease and related dementia

**DOI:** 10.1186/s13195-021-00818-3

**Published:** 2021-04-12

**Authors:** Yu-Jung Jenny Wei, Siegfried Schmidt, Cheng Chen, Roger B. Fillingim, M. Carrington Reid, Steven DeKosky, Laurence Solberg, Marco Pahor, Babette Brumback, Almut G. Winterstein

**Affiliations:** 1 Department of Pharmaceutical Outcomes and Policy, University of Florida College of Pharmacy, 1225 Center Drive, Health Professions Nursing Pharmacy Building, Room 3321, Gainesville, FL 32610 USA; 2 Center for Drug Evaluation and Safety, University of Florida, Gainesville, FL USA; 3 Department of Community Health and Family Medicine, College of Medicine, University of Florida, Gainesville, FL USA; 4 Pain Research and Intervention Center of Excellence, University of Florida, Gainesville, USA; 5 College of Dentistry, University of Florida, Gainesville, FL USA; 6 Division of Geriatrics and Palliative Medicine, Weill Cornell Medical College, New York, NY USA; 7 Department of Neurology, McKnight Brain Institute, University of Florida, Gainesville, FL USA; 8 NF/SG Veterans Health System, Malcom Randall VAMC, Geriatrics Research, Education, Clinical Center (GRECC), Gainesville, FL USA; 9 University of Florida College of Nursing, Gainesville, FL USA; 10 Department of Aging and Geriatric Research, Institute on Aging, University of Florida College of Medicine, Gainesville, FL USA; 11 Department of Biostatistics, University of Florida Colleges of Medicine and Public Health & Health Professions, Gainesville, FL USA; 12 Department of Epidemiology, University of Florida Colleges of Medicine and Public Health & Health Professions, Gainesville, FL USA

**Keywords:** Alzheimer’s disease and related dementias, Prescription opioids, Inappropriate prescribing

## Abstract

**Background:**

Pain is common among individuals with Alzheimer’s disease and related dementias (ADRD), and use of opioids has been increasing over the last decade. Yet, it is unclear to what extent opioids are appropriately prescribed for patients with ADRD and whether the appropriateness of opioid prescribing differs by ADRD status. The objective of this study is to compare the quality of opioid prescribing among patients with or without ADRD who have chronic noncancer pain.

**Methods:**

A nationally representative cohort study of Medicare beneficiaries aged 50 years or older who had chronic pain but who had no cancer, hospice, or palliative care from 2011 to 2015. Four indicators of potentially inappropriate opioid prescribing were measured in patients residing in communities (75,258 patients with and 435,870 patients without ADRD); five indicators were assessed in patients in nursing homes (NHs) (37,117 patients with and 5128 patients without ADRD). Each indicator was calculated as the proportion of eligible patients with inappropriate opioid prescribing in the year after a chronic pain diagnosis. Differences in proportions between ADRD and non-ADRD groups were estimated using a generalized linear model adjusting for covariates through inverse probability weighting.

**Results:**

Patients with ADRD versus those without had higher concurrent use of opioids and central nervous system–active drugs (community 44.1% vs 33.3%; NH 58.8% vs 54.1%, both *P* < 0.001) and no opioids or scheduled pain medications for moderate or severe pain (NH 60.1% vs 52.5%, *P* < 0.001). The ADRD versus non-ADRD group had higher use of long-term opioids for treating neuropathic pain in communities (21.7% vs 19.5%, *P* = 0.003) but lower use in NHs (26.9% vs 36.0%, *P* < 0.001). Use of strong or high-dose opioids when naive to opioids (community 1.5% vs 2.8%; NH 2.5% vs 3.5%) and use of contraindicated opioids (community 0.08% vs 0.12%; NH 0.05% vs 0.21%) were rare for either group.

**Conclusion:**

Potential inappropriate opioid prescribing in 2 areas of pain care was more common among patients with ADRD than among patients without ADRD in community or NH settings. Further studies aimed at understanding the factors and effects associated with opioid prescribing patterns that deviate from guidelines are warranted.

## Introduction

Pain is common among older adults with Alzheimer disease and related dementias (ADRD), with up to 60% of affected patients in communities and 80% of those in nursing homes (NHs) reporting regular pain [1]. Uncontrolled pain in ADRD contributes to poor physical function and mental disorders, notably depression, behavioral, and other psychiatric symptoms [2, 3].

Pharmacological treatment is commonly used to manage older adults’ pain [4]. Historical data have shown lower use of analgesics in patients with ADRD vs those without [5]. Memory decline and decreased communication skills among patients with cognitive impairment have been implicated in the underuse of analgesics [5, 6]. Findings of recent population-based studies, however, suggest that patients with ADRD were equally or more likely than patients without ADRD to receive analgesics for pain management [7–10]. The increasing analgesic use in patients with ADRD may result from increasing clinical awareness of the need for improved pain assessment and management in this patient population [7, 10].

Inappropriate medication prescribing practice is one of the primary reasons for uncontrolled pain [8, 10–12]. Opioids are commonly used to treat pain in older adults, with nearly 1 in 5 older adults filling at least 1 prescription opioid in 2015 [13]. To date, it remains unclear the extent to which opioids are being prescribed appropriately for older adults with chronic noncancer pain and whether the quality of opioid prescribing differs between those with or without ADRD. To address this question, we assessed and compared quality indicators of appropriate opioid prescribing practices commonly described in guidelines and published literature for the management of chronic pain among older adults with ADRD vs those without (Table 1). The selected quality indicators were assessed in both community and NH settings.

**Table 1:** Quality measures of potentially inappropriate opioid prescribing among older adults (≥ 50 years) with chronic noncancer pain

Measure domain	Indicators of inappropriate opioid prescribing	Operationalization with 2011–2015 Medicare and MDS 3.0 data		
		Denominator	Numerator	Exclusion
1. Opioids contraindicated for older adults	• Use of meperidine, propoxyphene, pentazocine, butorphanol, and nalbuphine	Older adults with chronic pain	Patients with contraindicated opioids	Cancer, hospice, or palliative care
2. Opioid-naïve patients	• Use of long-acting (LA) or extended-release (ER) prescription opioids for opioid-naïve patients	Patients with chronic pain who were naïve to opioids (i.e., no opioids in 6 months before an index chronic pain diagnosis [baseline])	Patients with LA/ER prescription opioids or using medications without evidence of drug tolerance (i.e., receive ≥ 60 daily MME) for a week or longer	
	• Use of high-dose prescription opioids for opioid-naïve patients		Patients with a daily dose of ≥ 90 MME	
	• Composite of any		Patients with LA/ER prescription opioids or with a daily dose of ≥ 90 MME	
3. Patients with neuropathic pain	• Long-term (> 90 days) use of opioids	Older adults with only neuropathic pain as the index diagnosis	Patients with long-term use of opioids	
		*Sensitivity analysis*: older adults with only neuropathic pain who had no musculoskeletal or idiopathic pain during the 6-month baseline		
4. Concurrent use of opioids and CNS depressants	• Concurrent use of opioids with any CNS drugs that Beers Criteria^1^ recommends against	Older adults with chronic pain and using prescription opioids	Patients with concurrent use of Beers Criteria^1^ CNS depressants for ≥ 7 days	
5. Patients with moderate to severe pain (NHs only)	• No prescription opioids within 30 days before or after reporting moderate to severe pain	Older adults with moderate to severe chronic pain	Patients with no prescription opioids within 30 days before or after the qualifying pain score	
	• No use of scheduled pain medication regimen in the 5 days before reporting moderate to severe chronic pain (defined based on MDS 3.0 pain assessment)		Patients with no scheduled pain medication regimen (defined based on MDS 3.0 item J0100A)	
	• Composite of any		Patients with no prescription opioids or no scheduled pain medications	

*Abbreviations*: *CNS* central nervous system, *MDS 3.0* Minimum Data Set, version 3.0, *MME* morphine milligram equivalent, *NHs* nursing homes

^1^American Geriatrics Society 2015 updated Beers criteria for potentially inappropriate medication use in older adults

## Methods

### Study design and source

We conducted a cohort study of a 5% random sample of Medicare beneficiaries linked to the Minimum Data Set, version 3.0 (MDS 3.0) from 2011 to 2015. Medicare data contain fee-for-service enrollees’ medical billing records for Parts A, B, and D (prescription drugs) and beneficiary-level sociodemographic characteristics, enrollment status, and presence of 27 chronic conditions, including ADRD [14]. The University of Florida Institutional Review Board approved and waived patient informed consent for this study.

The latest version of a federally mandated clinical assessment, MDS 3.0, collects data from all residents of Medicare- or Medicaid-certified NHs [15]. Most relevant to quality measures of opioid prescribing is MDS 3.0 Section J Health Condition, which documents self-reported pain intensity using numeric rating or verbal descriptor scale, supplemented with nursing staff–assessed pain using the checklist of nonverbal pain indicators for nonverbal residents, as well as the use of scheduled pain medication regimens. We used these pain-related data in MDS 3.0 to assess the appropriateness of opioid prescribing for moderate to severe pain among patients in NHs (Table 1).

### Study sample

The study sample included adults 50 years of age or older who had (1) at least 1 primary or secondary diagnosis of a chronic pain condition; (2) no diagnosis or procedures indicating cancer, hospice, or palliative care services; and (3) continuous enrollment in Medicare Parts A, B, and D for at least 18 months between 2011 and 2015. The 18-month period included a 6-month baseline before (for determining demographic and clinical characteristics, pain types, and history of opioid use) and a 12-month follow-up after a chronic pain diagnosis (for assessing the appropriateness of opioid prescribing). For each patient, we randomly selected one 18-month continuous enrollment period as an observation unit. We excluded patients with a health-maintenance-organization or an employer-sponsored insurance plan (owing to lack of complete encounters from in- and outpatient settings) during the 18-month period. The diagnostic and procedure codes for conditions and services considered in the sample selection are given in Supplement eTable 1.

We further categorized eligible patients based on residential status (community vs NH) because of the differences in patient characteristics and ADRD severity associated with this factor [16, 17]. Patients were classified as NH residents if they had at least 1 episode of a long NH stay (> 100 days, measured based on MDS 3.0 assessment dates) during the 12-month follow-up period [18]. The choice of using > 100 days to define long-stay NH residents is consistent with the definition set by the US Center for Medicare and Medicaid Services (CMS) [18]. Patients were classified as community dwellers if they had no or a short NH stay (≤ 100 days). We then created 4 community-dwelling cohorts and 5 NH cohorts, with each cohort corresponding to patients who were eligible for the denominator of a specific quality indicator (Supplement eFigure 1).

### Prescription opioid and other pain medications

We captured prescription opioids and other pain medications (including non-opioids analgesics and adjuvant treatments) using Medicare Part D files and measured drug use in the year after a chronic pain diagnosis (Supplement eTable 2). For opioids, we excluded injectable opioids used primarily in inpatient, rectal dosage forms that are rarely used, and buprenorphine in sublingual form and combined buprenorphine-naloxone products, which are used for addiction treatment. We converted the dose of each filled opioid prescription to daily morphine milligram equivalents (MMEs) by multiplying the quantity of opioids prescribed per day by the strength and MME conversion factor [19].

### Quality indicators of inappropriate opioid prescribing

Quality indicators of inappropriate opioid prescribing were defined based on core principles of clinical guidelines and published literature for the management of chronic noncancer pain in older adults (Supplement eTable 3). Four major quality indicators were selected and measured in the year after a chronic pain diagnosis: (1) use strong or high-dose (defined as ≥ 90 MME per day [20]) opioid regimen among opioid-naïve patients who had no prescription opioid use at baseline; (2) long-term use (≥ 90 days [21, 22]) of opioids for patients with neuropathic pain as the index diagnosis; (3) concurrent use of opioids with other central nervous system (CNS) depressant drugs for 7 or more days [23, 24]; and (4) use of opioids contraindicated for older adults [24–26]. An additional (fifth) indicator was measured only in NH patients based on MDS 3.0—the absence of opioid prescription or the absence of a scheduled pain treatment regimen within days of reporting moderate to severe pain [26, 27]. A detailed definition of each indicator is given in Table 1. Each indicator was calculated as the proportion of eligible patients with inappropriate opioid prescribing following the year after a chronic pain diagnosis.

### Statistical analysis

In both community and NH settings, we compared quality indicators between patients with versus without ADRD. Patients with ADRD were identified based on the Chronic Condition Data Warehouse flags as having at least one institutional (including inpatient, outpatient, skilled nursing facility, home healthcare, and hospice care) or office-based medical claims with any of 24 diagnostic codes for ADRD [14]. Because the ADRD and non-ADRD groups differed in many characteristics that may be associated with opioid prescribing practices, we used an inverse probability weighting (IPW) approach to balance differences in group characteristics that may bias outcome estimates, while retaining all study patients in the analysis [28]. In IPW, data from each patient were weighted by the inverse of the estimated probability of ADRD status conditional on measured baseline characteristics through a logistic regression model. Separate models were created to generate IPW weights using the characteristics of patient cohorts in communities (Table 2) and in NHs (Table 3). In the NH sample, in addition to characteristics measured from Medicare claims data, we also adjusted for three characteristics extracted from MDS 3.0 data—(1) Patient Health Questionnaire (PHQ)-9 depression symptoms (range 0–27), classified as no (0–4), mild (5–9), moderate (10–14), and severe depression (≥ 15) [29]; (2) activities of daily living (ADLs, range 0–28), classified into no (0–7), mild (8–14), moderate (15–21), and severe (≥ 21) dependence [30]; and (3) body mass index (BMI), calculated based on MDS-3.0 documented weight and height and classified into underweight (BMI < 18.5 kg/m^2^), normal (18.5–24.9), obsess (25.0–29.9), and overweight (≥ 30) [31]. We refrained from adjusting for MDS 3.0-assessed cognitive function and aggressive behaviors in the NH sample due to only a small proportion (< 5%, data not shown) of non-ADRD residents having moderate to severe cognitive function or aggressive behaviors. Adjustment of these two variables along with other characteristics would have produced extreme IPW weights, an indicative of violation of positivity assumption (i.e., probability of any patient having exposure [i.e., ADRD vs non-ADRD] is positive, nonzero with each stratum of covariate combination) [32].

**Table 2:** Demographic and clinical characteristics of community-dwelling older patients with chronic pain with or without ADRD stratified by cohort

Baseline characteristic^**a**^	Patients with chronic pain, %			Opioid-naive patients, %			Patients with neuropathic pain, %			Patients with opioid prescription, %		
	With ADRD	Without ADRD	*P*-value^d^	With ADRD	Without ADRD	*P*-value^d^	With ADRD	Without ADRD	*P*-value^d^	With ADRD	Without ADRD	*P*-value^d^
Total no.	75,258	435,870		48,182	304,009		9775	64,692		27,801	197,886	
**Age**, y												
Mean (SD)	79.3 (10.4)	70.9 (9.4)	< .001	80.4 (10.1)	72.1 (8.8)	< .001	76.5 (10.1)	69.7 (9.3)	< .001	77.8 (10.6)	69.2 (9.8)	< .001
50–64	9.4	19.3		7.5	13.1		12.5	23.2		11.9	27.2	
65–74	19.3	48.0		17.1	50.9		26.2	48.0		22.5	44.6	
75–84	36.3	24.7		36.4	27.0		38.1	22.6		36.1	21.7	
≥ 85	35.0	8.0		38.9	8.9		23.1	6.1		29.5	6.5	
**Female**	71.1	63.1	< .001	69.5	62.4	< .001	67.3	62.0	< .001	73.5	64.0	< .001
**Race/ethnicity**												
White	78.7	82.0	< .001	78.0	82.6	< .001	76.8	82.1	< .001	79.4	81.2	< .001
Black	11.7	10.4		11.3	9.1		12.4	10.3		12.3	12.3	
Others^b^	9.6	7.6		10.6	8.3		10.8	7.6		8.2	6.4	
**US region**												
Northeast	21.0	18.9	< .001	24.7	20.9	< .001	20.7	19.6	< .001	15.1	15.0	< .001
Midwest	21.7	24.5		21.5	24.7		19.9	23.9		22.0	24.3	
South	40.1	38.4		36.8	36.1		42.3	39.0		45.6	42.8	
West or other regions	17.2	18.2		17.1	18.3		17.1	17.5		17.2	17.8	
**Low-income subsidy**	44.0	34.5	< .001	41.2	28.5	< .001	46.6	35.7	< .001	47.9	42.3	< .001
**Tobacco or alcohol use**	6.7	6.5	.057	4.6	4.1	< .001	7.3	6.7	.021	9.3	9.5	.391
**Drug use disorder**	2.3	1.6	< .001	1.1	0.6	< .001	3.0	1.8	< .001	3.9	2.7	< .001
**Index pain diagnosis**^c^												
Musculoskeletal	88.8	86.7	< .001	89.4	87.4	< .001	24.5	19.7	< .001	88.0	86.3	< .001
Neuropathic	13.0	14.8	< .001	12.2	14.3	< .001	100.0	100.0	NA	14.2	15.2	< .001
Idiopathic	2.4	2.0	< .001	1.2	0.8	< .001	1.2	0.7	< .001	4.1	3.6	< .001
**Comorbidity affecting pain treatment**												
Cardiovascular disease	85.4	68.1	< .001	83.3	65.0	< .001	86.8	70.3	< .001	87.5	71.2	< .001
Pulmonary condition	52.9	40.7	< .001	47.9	35.9	< .001	53.5	41.0	< .001	59.2	46.5	< .001
Diabetes	39.8	34.3	< .001	38.0	32.4	< .001	59.4	47.7	< .001	42.6	36.7	< .001
Mental disorder	37.9	17.6	< .001	32.9	13.2	< .001	37.5	18.8	< .001	43.5	22.7	< .001
Gastrointestinal tract disorder	27.2	13.7	< .001	23.8	11.4	< .001	26.8	14.2	< .001	30.6	15.9	< .001
Urinary tract infection	24.6	9.4	< .001	22.1	8.1	< .001	22.3	10.0	< .001	26.9	10.7	< .001
Kidney disease	21.8	11.2	< .001	19.3	9.6	< .001	24.3	13.1	< .001	24.7	13.0	< .001
Fall or fracture	19.5	5.5	< .001	14.7	3.2	< .001	13.8	4.5	< .001	22.7	7.0	< .001
Neurodegenerative disease	13.7	3.5	< .001	12.6	2.7	< .001	15.9	4.7	< .001	15.0	4.3	< .001
Liver disease	6.1	4.3	< .001	5.2	3.5	< .001	6.6	4.6	< .001	6.9	5.1	< .001
**Health care utilization**												
Any hospitalization stay	25.0	8.2	< .001	20.5	5.1	< .001	21.4	7.9	< .001	28.7	10.6	< .001
Any ED visit	28.3	14.0	< .001	21.9	9.2	< .001	26.9	14.3	< .001	33.5	18.1	< .001
Any hospital surgical procedure	6.9	3.3	< .001	4.4	1.3	< .001	5.3	2.6	< .001	8.7	4.3	< .001

*Abbreviations*: *ADRD* Alzheimer disease and related dementias, *ED* emergency department

^a^Defined as the 6 months prior to the date of a randomly selected chronic pain diagnosis for each patient

^b^Included Hispanic, Asian, Pacific Islander, and Native American individuals

^c^Measured as primary or secondary diagnosis as the index diagnosis

^d^Statistical comparisons and *P*-values were calculated using *t*-tests for continuous variables and chi-square tests for categorical variables

**Table 3:** Demographic and clinical characteristics of older patients with chronic pain with or without ADRD residing in a nursing home stratified by cohort

Baseline characteristic^**a**^	Patients with chronic pain, %			Opioid-naive patients, %			Patients with neuropathic pain			Patients with opioid prescription, %			Patients with moderate to severe pain, %^**b**^		
	With ADRD	Without ADRD	*P*-value^g^	With ADRD	Without ADRD	*P*-value^g^	With ADRD	Without ADRD	*P*-value^g^	With ADRD	Without ADRD	*P*-value^g^	With ADRD	Without ADRD	*P*-value^g^
Total no.	37,117	5128		23,205	2761		2602	686		17,570	3180		10,971	2329	
**Age**, **y**															
Mean (SD)	82.2 (10.5)	73.1 (12.8)	< .001	82.6 (10.4)	73.8 (13.0)	< .001	77.3 (11.4)	69.2 (10.9)	< .001	81.6 (10.5)	72.9 (12.5)	< .001	80.9 (10.6)	72.7 (12.5)	< .001
50–64	7.1	28.2		6.8	26.8		15.2	34.5		7.6	27.8		8.5	28.7	
65–74	14.6	27.4		13.7	26.1		23.0	34.7		15.7	28.8		17.2	28.3	
75–84	29.8	21.0		29.4	21.3		31.0	21.0		30.5	21.0		31.2	20.8	
≥ 85	48.4	23.4		50.0	25.8		30.8	9.8		46.1	22.3		43.2	22.1	
**Female**	75.4	64.3	< .001	73.0	61.1	< .001	70.6	61.3	< .001	78.3	67.5	< .001	78.9	69.3	< .001
**Race/ethnicity**															
White	80.4	81.4	.205	78.6	80.3	.114	77.6	81.8	.082	83.1	82.7	.128	84.8	84.3	.662
Black	14.1	13.2		15.1	13.7		15.4	12.6		12.8	12.4		11.6	11.7	
Others^c^	5.5	5.4		6.3	5.9		7.0	5.6		4.1	4.9		3.7	4.0	
**US region**															
Northeast	23.2	18.8	< .001	27.6	22.2	< .001	23.1	19.8	< .001	17.2	16.3	< .001	19.3	17.5	< .001
Midwest	26.2	34.4		25.1	34.7		25.4	33.1		27.7	34.3		29.9	36.0	
South	40.0	33.1		37.1	30.9		38.7	33.2		44.3	35.5		40.7	32.7	
West or other regions	10.5	13.6		10.3	12.2		12.8	13.9		10.8	13.9		10.1	13.8	
**Low-income subsidy**	82.5	79.6	< .001	81.2	76.1	< .001	85.1	81.2	.018	82.5	79.9	< .001	83.9	81.5	.006
**Tobacco or alcohol use**	5.3	8.9	< .001	4.6	7.8	< .001	6.8	8.1	.259	6.5	9.6	< .001	7.3	9.4	< .001
**Drug use disorder**	1.7	3.0	< .001	1.1	1.5	.051	2.0	2.8	.217	2.5	3.9	< .001	2.8	4.0	.002
**Index pain diagnosis**^d^															
Musculoskeletal	92.5	86.4	< .001	93.6	88.1	< .001	13.1	10.1	.044	90.8	85.1	< .001	90.6	85.1	< .001
Neuropathic	6.0	11.8	< .001	5.6	11.3	< .001	100	100	NA	6.6	12.4	< .001	6.9	11.6	< .001
Idiopathic	3.0	3.7	.012	1.8	1.6	.551	0.7	0.7	.956	4.7	5.1	.281	4.6	5.7	.022
**Comorbidity affecting pain treatment**															
Cardiovascular disease	93.2	88.1	< .001	92.0	84.5	< .001	93.0	88.3	< .001	94.4	90.1	< .001	94.6	90.1	.025
Pulmonary condition	57.6	58.0	.565	54.1	51.5	.009	59.0	55.5	.130	62.1	61.3	.391	64.3	61.8	< .001
Diabetes	42.5	50.7	< .001	40.5	45.9	< .001	69.2	66.3	.169	44.8	53.2	< .001	46.0	53.6	< .001
Mental disorder	60.1	46.3	< .001	56.3	40.1	< .001	62.9	45.8	< .001	63.9	48.1	< .001	67.0	49.5	< .001
Gastrointestinal tract disorder	43.9	36.5	< .001	41.1	32.3	< .001	45.6	38.8	.003	46.3	38.3	< .001	47.0	38.9	< .001
Urinary tract infection	41.3	27.9	< .001	37.9	22.6	< .001	41.5	31.9	< .001	45.2	30.8	< .001	46.0	31.3	< .001
Kidney disease	26.7	28.6	.006	24.2	23.9	.732	32.9	33.4	.809	30.2	30.8	< .001	30.8	30.7	.943
Fall or fracture	24.0	16.7	< .001	19.8	12.3	< .001	18.9	10.6	< .001	28.5	19.4	< .001	28.5	18.7	< .001
Neurodegenerative disease	16.4	10.6	< .001	15.5	8.7	< .001	20.6	12.1	< .001	17.2	11.5	< .001	18.5	11.8	< .001
Liver disease	5.5	6.7	< .001	4.9	5.2	.548	6.8	7.3	.666	6.3	7.4	< .001	6.8	7.3	.423
**Health care utilization**															
Any hospitalization stay	31.7	29.9	.009	29.1	25.7	< .001	33.9	27.4	.003	35.9	32.5	< .001	37.7	32.4	< .001
Any ED visit	26.5	24.8	.009	22.3	19.2	< .001	26.6	25.5	.559	30.9	28.2	.003	31.5	27.9	< .001
Any hospital surgical procedure	7.6	9.7	< .001	6.2	7.2	.053	8.2	8.8	.642	10.0	11.4	.017	10.1	11.1	.174
**ADL dependence**^e^															
None	22.7	29.7	< .001	22.2	31.2	< .001	24.0	24.3	.207	23.3	26.8	< .001	26.5	28.5	.006
Mild	31.6	27.8		31.8	28.1		27.7	26.1		31.7	28.1		32.0	29.2	
Moderate	29.2	29.5		28.9	27.2		30.5	34.4		29.5	32.5		28.0	30.0	
Severe	16.5	13.0		17.0	13.5		17.8	15.2		15.5	12.7		13.5	12.3	
**PHQ-9 depression symptoms**^e^															
None	78.7	79.8	.247	80.2	81.3	.292	78.8	83.8	.027	76.4	77.7	.233	74.0	76.6	.017
Mild	14.7	14.2		13.8	13.5		15.1	11.4		16.3	15.6		17.7	16.4	
Moderate	4.9	4.6		4.6	4.1		4.2	4.0		5.2	5.1		5.8	5.5	
Severe	1.7	1.4		1.4	1.1		1.9	0.8		2.1	1.6		2.4	1.5	
**BMI**^e^															
Underweight	7.9	7.5	< .001	7.9	7.2	< .001	5.2	6.4	< .001	8.2	7.9	< .001	7.4	7.3	< .001
Normal weight	36.5	23.4		37.9	26.6		28.1	19.0		34.7	21.0		32.2	18.9	
Overweight	29.6	24.1		30.5	25.9		31.1	21.7		28.1	22.9		27.9	22.1	
Obese	25.9	45.0		23.6	40.2		35.6	52.9		28.9	48.1		32.4	51.7	
**Nursing home stay duration,** mean (SD), d^f^	326.3 (65.6)	263.8 (100.5)	< .001	325.0 (66.4)	261.7 (101.0)	< .001	316.0 (77.5)	254.9 (102.9)	< .001	323.3 (69.7)	256.3 (102.5)	< .001	331.6 (59.5)	268.0 (98.2)	< .001

*Abbreviations*: *ADRD* Alzheimer disease and related dementias, *ADL* activities of daily living, *BMI* body mass index (calculated as weight in kilograms divided by height in meters squared), *ED* emergency department, *MDS 3.0* Minimum Data Set, version 3.0, *PHQ-9* Patient Health Questionnaire-9

^a^Defined as the 6 months prior to the date of a randomly selected chronic pain diagnosis for each patient

^b^Defined as having ≥ 1 quarterly MDS 3.0 pain assessment with a numeric rating scale score of ≥ 4 or moderate or severe pain based on the verbal descriptor scale

^c^Included Hispanic, Asian, Pacific Islander, and Native American individuals

^d^Measured as primary or secondary diagnosis as the index diagnosis

^e^Measured based on the first observe quarterly MDS 3.0 assessment during 12 months after diagnosis of chronic pain, which was randomly selected per patient

^f^Measured during the entire 12 months after diagnosis of chronic pain, which was randomly selected per patient

^g^Statistical comparisons and *P*-values were calculated using *t*-tests for continuous variables and chi-square tests for categorical variables

We reported the weighted proportion of patients with chronic pain who received any pain medications, overall and by therapeutic classes (opioids, non-opioids, and adjuvants by therapeutic class) during the 12-month follow-up in ADRD or non-ADRD groups, by residential status. For each quality indicator and each setting, we also reported the weighted proportion of patients with inappropriate opioid prescribing between ADRD and non-ADRD groups. We estimated the proportion differences between groups and their 95% CIs using generalized linear models. All analyses were performed from April 2019 to April 2020 using SAS, version 9.4 (SAS Institute Inc). Statistical significance was set at *P* < 0.05, and all tests were 2-sided.

## Results

Of 553,373 Medicare beneficiaries with chronic pain identified, 75,258 patients with ADRD and 435,870 patients without ADRD were living in communities (Table 2), and 37,117 patients with ADRD and 5128 patients without ADRD were residing in NHs (Table 3). In both settings, compared with patients without ADRD, those with ADRD were older (mean [SD] age: in community, 79 [10] vs 71 [9] years; in NH, 82 [10] vs 73 [13] years; both *P* < .001) and were more likely to be female (community, 71% vs 63%; NH, 75% vs 64%; both *P* < .001). The proportion of nonwhite was higher in patients with vs without ADRD in communities (21% vs 18%, *P* < .001) but was similar in NHs (20% vs 19%; *P* = .205). After IPW, distributions of all measured baseline characteristics were well balanced between ADRD and non-ADRD groups in all patient cohorts in communities and NHs (Supplement eTables 4 and 5), with standardized mean differences for characteristics less than 0.1 (Supplement eFigures 2 and 3).

### Receipt of pain medications in patients with or without ADRD

After IPW, patients with ADRD were less likely than their non-ADRD counterparts to receive any prescription pain medication in the year after a chronic pain diagnosis in communities (66.6% vs 67.6%; difference, − 1.0% [95% CI, − 1.4 to − 0.7%]; *P* < .001) and NHs (64.5% vs 74.9%; difference, − 10.4% [95% CI, − 11.7 to − 9.1%]; *P* < .001) (Table 4). In both settings, analyses by therapeutic classes of analgesics showed lower use of opioids (community, 45.1% vs 48.1%; difference, − 3.0% [95% CI, − 3.4 to − 2.6%]; NH, 47.9% vs 60.7%; difference, − 12.8% [95% CI, − 14.2 to − 11.3%]; both *P* < .001) or lower use of non-opioids (community, 30.3% vs 32.7%; difference, − 2.4% [95% CI, − 2.8 to − 2.1%]; NH, 18.0% vs 21.9%; difference, − 3.9% [95% CI, − 5.1 to − 2.7%]; both *P* < .001) in patients with vs without ADRD. Among opioid users, the use of long-term opioids, however, varied by residential setting, with higher use in patients with ADRD in communities (41.2% vs 35.7%; difference, 5.4% [95% CI, 4.9 to 6.0%]; *P* < .001) and lower use in those residing in NHs (52.5% vs 58.2%; difference, − 5.7% [95% CI, − 7.6 to − 3.8%]; *P* < .001), when compared to their non-ADRD counterparts. A similar pattern was also observed for use of adjuvant analgesics.

**Table 4:** Weighted proportion of prescription pain medication use among older patients with chronic pain and with or without ADRD by residential setting

Medication	Community				Nursing home			
	Weighted proportion among patients with ADRD, %	Weighted proportion among patients without ADRD, %	Proportion difference (95% CI)^**a**^	***P***-value	Weighted proportion among patients with ADRD, %	Weighted proportion among patients without ADRD, %	Proportion difference (95% CI)^**a**^	***P***-value
**Any use of prescription pain medication**^b,c^	66.6	67.6	− 1.0 (− 1.4 to − 0.7)	< .001	64.5	74.9	− 10.4 (− 11.7 to − 9.1)	< .001
**Opioid**								
Any use	45.1	48.1	− 3.0 (− 3.4 to − 2.6)	< .001	47.9	60.7	− 12.8 (− 14.2 to − 11.3)	< .001
Long-term use of opioid^d^	41.2	35.7	5.4 (4.9 to 6.0)	< .001	52.5	58.2	− 5.7 (−7.6 to − 3.8)	< .001
**Non-opioid**								
Any use	30.3	32.7	− 2.4 (− 2.8 to − 2.1)	< .001	18.0	21.9	− 3.9 (− 5.1 to − 2.7)	< .001
**Adjuvant analgesic**								
Any use	35.9	32.7	3.1 (2.8 to 3.5)	< .001	33.2	40.9	− 7.6 (− 9.1 to − 6.2)	< .001
SNRI or tricyclic antidepressant	40.0	26.8	13.1 (12.7 to 13.5)	< .001	59.4	53.3	6.1 (4.6 to 7.6)	< .001
Anticonvulsant	21.1	17.7	3.4 (3.1 to 3.7)	< .001	21.9	27.2	− 5.3 (− 6.7 to − 4.0)	< .001
Skeletal muscle relaxant	14.2	15.6	− 1.4 (− 1.7 to − 1.2)	< .001	8.2	14.8	− 6.7 (− 7.7 to − 5.6)	< .001
**Topical analgesic**^e^	8.1	6.6	1.5 (1.3 to 1.7)	< .001	8.8	11.9	− 3.0 (− 4.0 to − 2.1)	< .001

*Abbreviations*: *ADRD* Alzheimer disease and related dementias, *SNRI* serotonin-norepinephrine reuptake inhibitor

^a^Proportion difference between patients with or without ADRD was estimated with generalized linear models with weight statement (to incorporate inverse probability weighting that balances differences in baseline characteristics between the ADRD and non-ADRD groups)

^b^Use of prescription pain medications was measured during the 12 months after diagnosis of chronic pain, which was randomly selected per patient

^c^A patient may have more than 1 type of drug combination during the 12-month observation period

^d^The proportion was calculated among opioid users

^e^Prescription topical analgesics included diclofenac and lidocaine

### Quality measure 1: contraindicated prescription opioids among chronic pain patients

Among patients with chronic pain, those with ADRD were less likely than their respective non-ADRD counterparts to receive meperidine, propoxyphene, or partial or mixed opioid agonists contraindicated for older adults, in communities (0.08% vs 0.12%; difference, − 0.04% [95% CI, − 0.06 to − 0.01%]; *P* < .001) or NHs (0.05% vs 0.21%; difference, − 0.2% [95% CI, − 0.3 to − 0.03%]; *P* < .001), though utilization was generally low (Table 5).

**Table 5:** Weighted proportion of inappropriate opioid prescribing practice for patients with or without ADRD by residential setting

Quality indicators of inappropriate opioid prescribing^a^	Community			***P***-value	Nursing home			***P***-value
	Weighted proportion among patients with ADRD, %	Weighted proportion among patients without ADRD, %	Proportion difference (95% CI)^**b**^		Weighted proportion among patients with ADRD, %	Weighted proportion among patients without ADRD, %	Proportion difference (95% CI)^**b**^	
**Use of opioids contraindicated for older adults with chronic pain**								
Use of meperidine, propoxyphene or partial or mixed opioid agonsists	0.08	0.12	− 0.04 (− 0.06 to − 0.01)	< .001	0.05	0.21	− 0.2 (− 0.3 to − 0.03)	< .001
**Opioid prescribing for opioid-naive patients**								
Use of long-acting opioid	0.49	0.62	− 0.13 (− 0.20 to − 0.06)	< .001	1.3	1.8	− 0.5 (− 1.0 to 0.04)	.070
Use of high-dose opioid	1.3	2.5	− 1.2 (− 1.3 to − 1.1)	< .001	1.9	2.6	− 0.7 (− 1.3 to − 0.05)	.035
Composite of either	1.5	2.8	− 1.3 (− 1.4 to − 1.1)	< .001	2.5	3.5	− 1.0 (− 1.7 to − 0.2)	.010
**Opioid prescribing for patients with neuropathic pain as the index diagnosis**								
Long-term use of opioid	21.7	19.5	2.2 (1.3 to 3.1)	.003	26.9	36.0	− 9.2 (− 13.6 to − 4.7)	< .001
**Sensitivity analysis: for patients with neuropathic pain without baseline musculoskeletal or idiopathic pain**								
Long-term use of opioid	12.1	10.4	1.8 (0.6 to 2.9)	< .001	25.7	33.1	− 7.5 (− 12.1 to − 2.8)	< .001
**Concurrent use of opioid and CNS depressant for opioid users**								
With any qualifying CNS depressant^c^	44.1	33.3	10.8 (10.2 to 11.4)	< .001	58.8	54.1	4.7 (2.8 to 6.6)	< .001
With benzodiazepine	19.5	15.3	4.2 (3.7 to 4.6)	< .001	21.0	23.2	− 2.2 (− 3.8 to − 0.6)	< .001
With SSRI or TCA	26.8	19.6	7.2 (6.7 to 7.7)	< .001	39.1	38.2	0.96 (− 0.9 to 2.8)	.307
With antipsychotic	10.4	4.6	5.8 (5.4 to 6.1)	< .001	26.2	11.4	14.8 (13.5 to 16.1)	< .001
With nonbenzodiazepine	9.5	8.2	1.3 (1.0 to 1.7)	< .001	6.7	9.7	− 3.0 (− 4.1 to − 1.9)	< .001
**Opioid or other scheduled analgesic regimen for moderate to severe pain**^d^								
No use of prescription opioid^e^	ND	ND	ND	ND	32.7	24.6	8.1 (6.1 to 10.1)	< .001
No use of scheduled pain medication^f^	ND	ND	ND	ND	47.8	43.1	4.7 (2.4 to 7.0)	< .001
Composite of either	ND	ND	ND	ND	60.1	52.5	7.6 (5.3 to 9.9)	< .001

*Abbreviations*: *ADRD* Alzheimer disease and related dementias, *CNS* central nervous system, *MDS 3.0* Minimum Data Set, version 3.0, *MME* morphine milligram equivalent, *ND* not determined, *SNRI* serotonin-norepinephrine reuptake inhibitor, *SSRI* selective serotonin reuptake inhibitor, *TCA* tricyclic antidepressant

^a^Measured during the 12 months after diagnosis of chronic pain randomly selected per patient

^b^Proportion difference between ADRD and non-ADRD groups was estimated with generalized linear models along with weight statement (to incorporate inverse probability weighting that balances differences in baseline characteristics between the ADRD and non-ADRD groups)

^c^CNS-active drugs included antipsychotics, benzodiazepine, nonbenzodiazepine or hypnotics, tricyclic antidepressants, and SNRIs

^d^Defined as having at least 1 quarterly MDS 3.0 pain assessment with a numeric rating scale score of 4 or more, or moderate or severe pain based on a verbal descriptor scale

^e^Measured as having at least 1 quarterly MDS 3.0 moderate to severe pain score without prescription opioids dispensed within 30 days before and after the MDS 3.0 pain assessment

^f^Measured as having at least 1 quarterly MDS 3.0 moderate to severe pain score that had no scheduled pain medications (assessed in MDS 3.0 Section J)

### Quality measure 2: strong or high-dose opioids for opioid-naive patients

Among patients with chronic pain who had no opioids at baseline, those with ADRD were less likely than their respective non-ADRD counterparts to receive long-acting or high-dose prescription opioids in communities (1.5% vs 2.8%; difference, − 1.3% [95% CI, − 1.4 to − 1.1%]; *P* < .001) or NHs (2.5% vs 3.5%; difference, − 1.0% [95% CI, − 1.7 to − 0.2%]; *P* = .010) (Table 5).

### Quality measure 3: long-term opioid use for neuropathic pain

Among patients who had neuropathic pain as the index diagnosis, those with ADRD were more likely than their non-ADRD counterparts to have long-term use of prescription opioids in communities (21.7% vs 19.5%; difference, 2.2% [95% CI, 1.3 to 3.1%]; *P* = .003) (Table 5). By contrast, in NHs, patients with ADRD and neuropathic pain were less likely than their non-ADRD counterparts to have long-term opioid use (26.9% vs 36.0%; difference, − 9.2% [95% CI, − 13.6 to − 4.7%]; *P* < .001) (Table 5). Similar results were observed in sensitivity analyses restricted to individuals with neuropathic pain who had no history of musculoskeletal or idiopathic pain at baseline.

### Quality measure 4: concurrent use of opioids and CNS drugs

Among patients with chronic pain who received prescription opioids, those with ADRD (vs their non-ADRD counterparts) had higher concurrent use of prescription opioids and other CNS-active drugs against guideline recommendations in communities (44.1% vs 33.3%; difference, 10.8% [95% CI, 10.2 to 11.4%]; *P* < .001) and NHs (58.8% vs 54.1%; difference, 4.7% [95% CI, 2.8 to 6.6%]; *P* < .001) (Table 5). This pattern was similar across different CNS drug classes in communities, with the largest between-group difference in concurrent use of opioids and antidepressants (including selective serotonin reuptake inhibitors [SSRIs] or tricyclic antidepressants [TCAs]) (difference, 7.2% [95% CI, 6.7 to 7.7%]; *P* < .001), followed by combined use with antipsychotics (difference, 5.8% [95% CI, 5.4 to 6.1%], *P* < 0.001) and benzodiazepines (difference, 4.2% [95% CI, 3.7 to 4.6%]; *P* < .001). In NHs, concurrent opioid and antipsychotic use was significantly higher (26.2% vs 11.4%; difference, 14.8% [95% CI, 13.5 to 16.1%]; *P* < .001), but lower combined use of opioids with benzodiazepine (21.0% vs 23.2%; difference, − 2.2% [95% CI, − 3.8 to − 0.6%]; *P* < .001) or nonbenzodiazepine (6.7% vs 9.7%; difference, − 3.0% [95% CI, − 4.1 to − 1.9%]; *P* < .001) was observed in patients with ADRD versus without. No difference was observed in the combined use of opioids with SSRIs or TCAs between ADRD and non-ADRD groups (39.1% vs 38.2%; difference, 0.96% [95% CI, − 0.9 to 2.8%]; *P* = .307).

### Quality measure 5 (NH only): opioid prescribing for moderate to severe pain

Among NH residents diagnosed as having chronic pain with at least 1 episode of moderate to severe pain during the year after a chronic pain diagnosis, those with ADRD were less likely than those without ADRD to have opioids prescribed during the 30 days before or after reporting moderate to severe pain, or to have a scheduled pain medication regimen in 5 days before the pain episode (60.1% vs 52.5%; difference, 7.6% [95% CI, 5.3–9.9%]; *P* < .001) (Table 5).

## Discussion

In this nationally representative study of older Medicare patients with chronic noncancer pain between 2011 and 2015, we found differences in adherence to current pain guidelines between patients with and without ADRD, but the magnitude and direction of the differences varied across the indicators of potentially inappropriate opioid prescribing. Notably, of the five indicators, two measures—concurrent use of prescription opioids and other CNS-active drugs and no scheduled opioids for moderate or severe pain—were more common among patients with ADRD than among patients without ADRD in community or NH settings. The other three measures of potentially inappropriate prescribing were similar or lower in patients with ADRD than in those without in either setting.

The concurrent use of prescription opioids and CNS-active drugs was prevalent (between 33 and 59%) in our study population residing in the community or NH setting. The estimated prevalence of concurrent opioid-CNS drug use echoes recent studies, suggesting a rise in CNS polypharmacy that involved opioids in older adults and in patients with dementia [33, 34]. A potential reason for our observed higher concurrent opioid-CNS drug use in ADRD may be its non-cognitive neuropsychiatric symptoms (NPS) (e.g., agitation, psychosis, depression, anxiety, and sleep disturbance) [35], and thus a greater perceived need for psychotropic medications [36]. While the use of psychotropics in ADRD is controversial due to concerns over safety associated with these drugs [37], antipsychotics are indicated for aggression and psychosis and antidepressants are for major depression and anxiety. Literature has documented the safety of concurrent use of opioids and benzodiazepines in older populations with or without ADRD [38–40]. However, to our best knowledge, no population-based studies have examined benefits and harms associated with concurrent use of opioids and antipsychotics or antidepressants, the most common drug combination in patients with ADRD observed by the present study. Given the high prevalence of ADRD patients who had comorbid chronic pain and NPS symptoms [2], future research is needed to identify risk factors and effects of potentially inappropriate concurrent opioid-psychotropic use on patient outcomes.

Among NH residents who reported moderate or severe pain, we observed that over half had no opioid prescribing nor scheduled pain medication regimen within several days of the pain reporting, with a higher proportion seen in patients with ADRD than those without. Our finding echoes previous studies, underscoring the long-standing concern on potential undertreatment of pain in patients with ADRD [7, 41]. It seems that proactive treatment with scheduled pain medications, including opioids, was less common in patients with ADRD than those without. The underlying causes for this disparity are unclear and may be related to health providers’ knowledge of and attitudes toward prescribing opioids for patients with ADRD [42–44]. Recent literature suggests over half of primary care doctors were uncertain about the safety of using opioids to treat pain in dementia patients, and many disagreed with prescribing analgesics regularly, even if this approach is considered the optimal treatment of pain [42]. Our results highlight that many NH residents with moderate or severe pain, particularly those with ADRD, might be at high risk of having their chronic pain undertreated.

Our study observed one in four community-dwelling older adults with neuropathic pain receiving long-term opioids in patients with or without ADRD. Our estimate is consistent with literature indicating that 22.7% of patients with dementia and polyneuropathy receive long-term opioid therapy [45]. Clinical guidelines often list opioids as a later-line treatment for neuropathic pain after failure of adjuvant and non-opioid therapy [46]. No guideline has endorsed long-term opioid use owing to limited evidence of efficacy, opioid dependency, and overdose concern [45]. Notably, we observed an opposite pattern in NHs, with lower use of long-term opioids among patients with than those without ADRD. The observed findings may be explained by differences in clinician specialties, with general or family medicine physicians being more likely to provide treatment for patients in communities, whereas geriatricians or advanced practitioners (nurse practitioners and physician assistants) being more likely to provide treatment in NHs [47].

The present study also compared the prevalence of receiving any prescription pain medications during the year after a chronic pain diagnosis and found a small difference between community-dwelling patients with and without ADRD (66.6% vs 67.6%). While the difference was statistically significant largely due to our large sample size, the magnitude of the difference was too small to indicate any clinical significance. Our estimate in the community population is consistent with that of a recent population-based study, suggesting that patients with ADRD were as likely to receive pain treatment as patients without ADRD in the community [7]. In NHs, we also observed 64.5% of residents with ADRD receiving pain treatment, although the figure was lower than that (74.9%) of residents without ADRD. The discrepancy in the use of pain treatment between NH residents with and without ADRD may be explained by differences in pain severity, cognitive function, and communication ability [9, 48]. The loss of verbal communication skills likely occurs among patients in the late stages of ADRD, leading to great difficulties in detecting pain [12]. Our estimate of pain medication use in the NH population is more aligned with data of recent studies [49] than those of earlier research [50].

### Implications

Our study has important implications for clinical and research purposes. Clinically, the use of prescription pain therapy, including opioids, among patients with ADRD has increased from 30–56% between 2006 and 2010 [41, 50, 51] to 67–90% between 2011 and 2016, observed in this and a recent study [49]. Such increase may reflect great improvements in awareness of pain assessment and management for older adults with ADRD, which have been emphasized for years by governments [52] and professional societies [24–26, 52]. Regarding opioid prescribing quality, clinical recommendations for avoiding strong or high-dose opioids for opioid-naïve patients and contraindicated opioids for older adults appear to translate well into clinical practice, with only 4% of older adults with or without ADRD receiving such inappropriate prescribing practices. Contrary to what the guidelines suggest, many patients with ADRD received opioids concurrently with other CNS drugs or received no scheduled opioids for moderate or severe pain. These discrepancies could be explained by many reasons, including time lag in adapting, lack of awareness, and disagreement on guidelines [53], which rely on evidence largely from cognitively intact older adults [12]. From research perspectives, it remains unclear whether these deviated opioid prescribing practices are associated with outcomes of patients with ADRD. Perhaps, the fundamental question that ought to be answered is whether opioids are safe, especially if used concurrently with psychotropic CNS drugs for neuropsychiatric symptoms (NPSs), which affects 95% of patients with ADRD [35]. These questions need to be addressed with considerations of limited treatment options available for NPSs, shortened life expectancy [54], and health outcomes (e.g., pain control, physical independence) that are attainable and desirable in patients with ADRD [55].

### Strengths and limitations

The Medicare claims data lacked information on some important elements, such as prior pain management experience (e.g., response and tolerance to opioids) and medical notes (e.g., drug and disease contraindications to opioids) that may justify deviations from guidelines. Although lacking these data may have hindered our ability to assess the quality of opioid prescribing, we mitigated this issue by balancing characteristics derived from Medicare claims or MDS 3.0 data, thus achieving comparison groups with presumably similar distributions of these factors that may explain deviations from guidelines. Analyses in a specific residential setting also helped reduce the heterogeneity of patient characteristics when comparing quality measures between patients with ADRD or without. Second, baseline depression and functional ability measured using MDS 3.0 were accounted for in the NH sample but not the community sample due to lack of information on these variables. Third, Medicare data do not detail the indication for which a drug was prescribed, which creates difficulty in determining the type of pain condition (e.g., neuropathic or nociceptive pain) for which the opioids were prescribed among patients with multiple co-existing pain conditions. Fourth, while MDS 3.0 used validated tools (i.e., numerical rating scale or verbal descriptor scale) to capture self-reported pain from NH patients who are capable of communication, these tools may not completely capture pain severity from patients with ADRD, particularly those in later stages where memory and communication ability is deteriorating [12]. Fifth, our results are derived from Medicare fee-for-service beneficiaries and cannot be generalized to those with Medicare Advantage or the non-Medicare population. Sixth, the assessed quality indicators of opioid prescribing may act against each other. For example, not prescribing opioids for patients with moderate to severe pain violates one guideline recommendation, but such practice may be necessary to avoid opioid use with other existing CNS drugs. Finally, the study used data prior to 2016, and it is unclear to what extent the 2016 Centers for Disease Control and Prevention guideline has shaped the quality of opioid prescribing in patients with ADRD and chronic pain. Studies exploring the impact of this more recent federal guidance are needed.

## Conclusions

Potential inappropriate opioid prescribing in 2 of 5 areas of pain care was more common among patients with ADRD than among patients without ADRD in community or NH settings between 2011 and 2015. Further studies exploring determinants and health outcomes associated with opioid prescribing in identified areas of pain care among patients with ADRD are warranted.

## Supplementary Information

**Additional file 1: Table S1**. *ICD-9-CM* Codes and Procedures for Disease Conditions and Service Care Considered in the Study. **Table S2**. Medications of Interest in This Study. **Table S3**. Current and Recent Guideline and Consensus Documents on Opioid Prescribing for Older Adults with Noncancer pain. **Table S4**. Characteristics of Community-Dwelling Patients with Chronic Pain With or Without ADRD Using Inverse Propensity Weighting, by Cohort. **Table S5**. Characteristics of Nursing Home Residents Who Have Chronic Pain With or Without ADRD Using Inverse Propensity Weighting, by Cohort. **Figure S1**. Flowchart of Included Patients. **Figure S2**. Absolute Standardized Differences for Baseline Demographic and Clinical Characteristics of Community-Dwelling Patients With Chronic Pain With or Without ADRD in the Original Population and After Inverse Probability Weighting. **Figure S3**. Absolute Standardized Differences for Baseline Demographic and Clinical Characteristics of Nursing Home Residents Who Had Chronic Pain With or Without ADRD in the Original Population and After Inverse Probability Weighting.

## Data Availability

The data that support the findings of this study are available from the Centers for Medicare and Medicaid Services but restrictions apply to the availability of these data, which were used under license for the current study, and so are not publicly available.
