# Supplementary material for: Quality of opioid prescribing in older adults with or without Alzheimer disease and related dementia
Source: Alzheimers Res Ther. 2021 Apr 12;13:78. doi: 10.1186/s13195-021-00818-3 (PMC8061026; doi:10.1186/s13195-021-00818-3)

**Supplementary Online Content**

**eTable 1.** *ICD-9-CM* Codes and Procedures for Disease Conditions and Service Care Considered in the Study

**eTable 2.** Medications of Interest in This Study

**eTable 3.** Current and Recent Guideline and Consensus Documents on Opioid Prescribing for Older Adults with Noncancer pain

**eTable 4.** Demographic and Clinical Characteristics of Community-Dwelling Patients with Chronic Pain With or Without ADRD Using Inverse Propensity Weighting, by Cohort

**eTable 5.** Demographic and Clinical Characteristics of Nursing Home Residents Who Have Chronic Pain With or Without ADRD Using Inverse Propensity Weighting, by Cohort

**eFigure 1.** Flowchart of Included Patients

**eFigure 2**. Absolute Standardized Differences for Baseline Demographic and Clinical Characteristics of Community-Dwelling Patients With Chronic Pain With or Without ADRD in the Original Population and After Inverse Probability Weighting

**efigure 3.** Absolute Standardized Differences for Baseline Demographic and Clinical Characteristics of Nursing Home Residents Who Had Chronic Pain With or Without ADRD in the Original Population and After Inverse Probability Weighting

**eTable 1. *ICD-9-CM* codes and Procedures for Disease, Condition, and Service Care Considered in the Study**

| **Disease, Condition, or Service Care** | ***ICD-9-CM* codes or procedures** | **Algorithm** |
| --- | --- | --- |
| ADRD | 331.x, 331.2, 331.7, 290.0-290.4x, 294.x (exclude 294.9), 797 | At least 1 inpatient, SNF, HHA, HOP or carrier claim with disease code |
| Chronic pain |  |  |
| Musculoskeletal | 274.x, 710.x-729.x (exclude 723.4, 724.3, 724.4, 729.1, 729.2) | At least 1 inpatient, SNF, HHA, HOP, or carrier claim with disease code |
| Neuropathic | 053.1x, 249.6, 250.6, 307.89, 336.x, 337.x, 338.0, 340, 350.x, 351.x, 352.1, 353.x-355.x, 357.1, 357.2-357.4, 357.8, 357.9, 723.4, 724.3, 724.4, 729.1, 729.2 |  |
| Idiopathic | 338.2, 338.4, 780.96 |  |
| Cancer diagnosis | CCS11-CCS43 | HCUP CCS for *ICD-9-CM* |
| Hospice care | Admission date of hospice claims | At least 1 hospice claim |
| Palliative Care | DX: V 66.7  Provide specialty code: 17 | At least 1 inpatient, SNF, HHA, HOP, carrier, or DME claim with disease code; or at least 1 inpatient, SNF, HHA, HOP with provider specialty code |
| Tobacco use or alcohol disorder | 305.1, 649.0x, 989.84;  291.x, 303.x, 305.0x, 357.5, 425.5, 535.3x, 571.0-571.3, 760.71, 980.0, V65.42, V79.1, E860.0 | At least 1 inpatient, SNF, HHA, HOP, carrier, or DME claim with disease code |
| Drug use disorder | 292.x, 304.x, 305.x (exclude 305.0x, 305.1), 648.3x, 655.5x, 760.72, 760.73, 760.75, 779.5, 965.0x | At least 1 inpatient, SNF, HHA, HOP, carrier, or DME claim with disease code |
| **Comorbidities associated with pain or pain management** |  |  |
| Cardiovascular disease | Heart valve disorders (CCS 96), essential hypertension (CCS 98), hypertension with complications and secondary hypertension (CCS 99), coronary atherosclerosis and other heart disease (CCS 101), pulmonary heart disease (CCS 103), cardiac dysrhythmias (CCS 106), congestive heart failure; nonhypertensive (CCS 108), acute cerebrovascular disease (CCS 109), occlusion or stenosis of precerebral arteries (CCS 110), other and ill-defined cerebrovascular disease (CCS 111), peripheral and visceral atherosclerosis (CCS 114) | HCUP CCS for *ICD-9-CM* |
| Pulmonary condition | Pneumonia (except that caused by tuberculosis or sexually transmitted disease) (CCS 122), acute bronchitis (CCS 125), other upper respiratory infections (CCS 126), chronic obstructive pulmonary disease and bronchiectasis (CCS 127), asthma (CCS 128), pleurisy; pneumothorax; pulmonary collapse (CCS 130), respiratory failure; insufficiency; arrest (CCS 131), other lower respiratory disease (CCS 133) |  |
| Diabetes | Diabetes mellitus without complication (CCS 49), diabetes mellitus with complications (CCS 50) |  |
| Mental disorder | Anxiety disorders (CCS 651), mood disorders (CCS 657), schizophrenia and other psychotic disorders (CCS 659) |  |
| Gastrointestinal tract disorder | Gastrointestinal hemorrhage (CCS 153), other gastrointestinal disorders (CCS 155), digestive congenital anomalies (CCS 214) |  |
| Urinary tract infection | CCS 159 |  |
| Kidney disease | Nephritis; nephrosis; renal sclerosis (CCS 156), acute and unspecified renal failure (CCS 157), chronic kidney disease (CCS 158), other diseases of kidney and ureters (CCS 161) |  |
| Falls or fractures | Pathological fracture (CCS 207), fracture of neck of femur (hip) (CCS 226), skull and face fractures (CCS 228), fracture of upper limb (CCS 229), fracture of lower limb (CCS 230), other fractures (CCS 231), e-codes: fall (CCS 2603). |  |
| Neurodegenerative disease | Parkinson disease (CCS 79), other hereditary and degenerative nervous system conditions (CCS 81) |  |
| Liver disease | CCS 151 |  |
| Hospital surgical procedure | Surgery Flags Software for *ICD-9-CM* created by AHRQ^1^ | At least inpatient or HOP claim with procedure code |

Abbreviations: ADRD, Alzheimer disease related dementias; AHRQ, Agency for Healthcare Research and Quality; CCS, Clinical Classification Software; DME, Duration Medical Equipment; HCUP, Healthcare Cost and Utilization Project; HHA, home health agency; HOP, hospital outpatient *ICD-9-CM*, *International Classification of Diseases, Ninth Revision, Clinical Modification*; SNF, skilled nursing facility.

^1^<https://www.hcup-us.ahrq.gov/toolssoftware/surgflags/surgeryflags.jsp>

**eTable 2. Medications of Interest in This Study**

| **Drug Class** | **Drugs** |
| --- | --- |
| **Prescription opioid** | Buprenorphine (exclude sublingual form and buprenorphine-naloxone combinations), butorphanol, codeine, dihydrocodeine, fentanyl, hydrocodone, hydromorphone, levorphanol, meperidine, methadone, morphine, opium, oxycodone, oxymorphone, pentazocine, and tramadol |
| Long-acting opioid | Hydromorphone, morphine, oxycodone, oxymorphone, tapentadol, and tramadol in extended release dosage forms, buprenorphine patch, fentanyl transdermal system, levorphanol, and methadone |
| Short-acting opioid | hydrocodone, hydromorphone, morphine, oxymorphone, oxycodone, tapentadol and tramadol in immediate release dosage forms, codeine, transmucosal and nontransdermal fentanyl formulations, and non-transdermal buprenorphine formulations |
| **Prescription nonopioid analgesics** |  |
| Analgesic/antipyretic | Acetaminophen, ziconotide |
| Nonsteroidal anti-inflammatory drug | Celecoxib, diclofenac potassium/sodium, etodolac, fenoprofen, flurbiprofen, ibuprofen, indomethacin, ketoprofen, ketorolac, meclofenamate, mefenamic acid, meloxicam, nabumetone, naproxen, oxaprozin, piroxicam, sulindac, tolmentin, aspirin, diflunisal, and salsalate |
| **Adjuvant analgesics** |  |
| Tricyclic antidepressant | Amitriptyline, imipramine, doxepin, clomipramine, trimipramine, nortriptyline, desipramine, amoxapine, and protriptyline |
| Serotonin-norepinephrine reuptake inhibitor | Duloxetine, milnacipran, venlafaxine, desvenlafaxine, and levomilnacipran |
| Anticonvulsant | Gabapentin, pregabalin, carbamazepine, lacosamide, oxcarbazepine, topiramate, and valproate |
| Muscle relaxant | Baclofen, cyclobenzaprine, tizanidine, chlorzoxazone, carisoprodol, dantrolene, metaxalone, methocarbamol, orphenadrine, and diazepam |
| **Central nervous system drugs not to prescribed with opioids suggested by the 2015 Beers criteria** |  |
| Antipsychotic | Aripiprazole, asenapine, brexpiprazole, cariprazine, chlorpromazine, clozapine, droperidol, fanapt, fluphenazine, haloperidol, iloperidone, loxapine, lurasidone, molindone, olanzapine, paliperidone, perphenazine, pimozide, prochlorperazine, quetiapine,  risperidone, thioridazine, thiothixene, trifluoperazine, ziprasidone |
| Selective serotonin reuptake inhibitor | Citalopram, escitalopram, fluoxetine, fluvoxamine, paroxetine, sertraline, vilazodone |
| Benzodiazepine | Alprazolam, estazolam, lorazepam, oxazepam, temazepam, triazolam, midazolam, chlordiazepoxide, clonazepam, clorazepate, diazepam, flurazepam, quazepam, clobazam |
| Nonbenzodiazepine or hypnotic | Buspirone, meprobamate, eszopiclone, zaleplon, zolpidem, belsomra |

**eTable 3. Current and Recent Guideline and Consensus Documents on Opioid Prescribing for Older Adults with Noncancer pain**

| **Type of pain care** | **Guideline recommendation** |
| --- | --- |
| Opioid-naive patients | **2009 AGS guideline; 2015 AMDG guideline***:* Avoid long-acting/extended-released opioids for opioid-naive patients or patients with no evidence of opioid tolerance, defined as receiving an opioid dosage of at least 60 mg morphine milligram equivalent (MME) daily for a week or longer.  **2009 AGS guideline; 2016 CDC guideline:** Avoid high-dose opioids for opioid-naïve patients; clinicians should start opioids at a low dose in new opioid users. |
| Patients with neuropathic pain | **2015 AMDG guideline**: The use of opioids for nonspecific low back pain, headaches, and fibromyalgia is not supported by evidence.  **2015 AMDG guideline**: There is no evidence from randomized trials to support the use of opioids for fibromyalgia, despite some observational studies showing that strong opioids are used in fibromyalgia patients. |
| Patients with moderate to severe pain | **2009 AGS guideline**: All patients with moderate to severe pain, pain-related functional impairment, or diminished quality of life due to pain should be considered for opioid therapy.  **2009 AGS guideline**: Use scheduled (around-the-clock) pain medications for patients with moderate to severe pain, or frequent or continuous pain on a daily basis.  **2012 ACEP guideline**: Opioids should be reserved for more severe pain or pain refractory to other analgesics.  **2012 AAPM guideline**: Clinicians may consider opioids if chronic noncancer pain is moderate or severe.  **2015 AMDG guideline**: Reserve opioids for moderate to severe acute pain. If used, utilize the lowest possible dose as part of a multimodal regimen, including NSAIDs, acetaminophen, and nonpharmacologic therapies, unless contraindicated. |
| Avoid combination use of opioids and other CNS-active drugs | **2015 Beers Criteria***:* Avoid prescribing opioids concurrently with CNS drugs, including antipsychotics; benzodiazepines; nonbenzodiazepine, benzodiazepine receptor agonist hypnotics; tricyclic antidepressants; selective serotonin reuptake inhibitors owing to risk of falls and fractures |
| Avoid opioids contraindicated for older adults | **2012/2015 Beers Criteria and 2015 AMDG guideline**: Avoid meperidine because the metabolite, normeperidine, is toxic to the CNS and can cause seizures, mood alterations, and confusion in older patients, especially those with renal impairment.  **2015 AMDG guideline; 2015 Beers Criteria:** Avoid agonist-antagonist opioids among older adults owing to the risk of CNS adverse effects, including confusion and hallucinations.  **2015 Beers Criteria**: Avoid skeletal muscle relaxants because most of the drugs are poorly tolerated by older adults owing to anticholinergic adverse effects, sedation, and increased risk of fractures. |
| 2009 American Geriatrics Society (AGS) guideline  2010 FDA Drug Safety Communication: FDA recommends against the continued use of propoxyphene  2010 Recommendations for the Pharmacological Management of Neuropathic Pain (PMNP): An Overview and Literature Update  2012 American Academy of Pain Medicine (AAPM) guideline  2012 The American College of Emergency Physicians (ACEP) guideline  2015 The Washington State Agency Medical Directors’ Group (AMDG) Guideline  2012/2015 Beer criteria | |

Abbreviations not indicated in the table: CDC, Centers for Disease Control and Prevention; CNS, central nervous system; FDA, US Food and Drug Administration; NSAIDs, nonsteroidal anti-inflammatory drugs.

**eTable 4. Demographic and Clinical Characteristics of Community-Dwelling Patients with Chronic Pain With or Without ADRD Using Inverse Propensity Weighting, by Cohort**

| **Baseline characteristic^a^** | **Patients with chronic pain, %** | | **Opioid-naive patients, %** | | **Patients with neuropathic pain, %** | | **Patients with prescription opioids, %** | |
| --- | --- | --- | --- | --- | --- | --- | --- | --- |
|  | **With ADRD** | **Without ADRD** | **With ADRD** | **Without ADRD** | **With ADRD** | **Without ADRD** | **With ADRD** | **Without ADRD** |
| **Total, No.** | 72,572 | 441,200 | 45,692 | 307,658 | 9,565 | 65,311 | 32,564 | 209,636 |
| **Age**, y |  |  |  |  |  |  |  |  |
| Mean (SD) | 72..5 (10.4) | 72.1 (10.0) | 73.9 (9.9) | 73.2 (9.5) | 70.9 (10.0) | 70.6 (9.7) | 70.8 (10.5) | 70.3 (10.3) |
| 50-64 | 22.3 | 17.7 | 15.9 | 12.3 | 26.8 | 21.8 | 29.1 | 25.0 |
| 65-74 | 38.8 | 43.4 | 41.3 | 45.9 | 40.4 | 44.9 | 37.6 | 41.3 |
| 75-84 | 26.4 | 26.4 | 28.8 | 28.2 | 24.2 | 24.6 | 23.3 | 23.7 |
| ≥85 | 12.5 | 12.5 | 14.0 | 13.6 | 8.5 | 8.7 | 10.0 | 10.0 |
| **Female** | 64.0 | 64.4 | 63.1 | 63.4 | 63.6 | 62.8 | 65.3 | 65.5 |
| **Race/ethnicity** |  |  |  |  |  |  |  |  |
| White | 81.2 | 81.5 | 81.7 | 81.9 | 80.8 | 81.3 | 80.8 | 81.0 |
| Black | 10.8 | 10.5 | 9.6 | 9.3 | 11.3 | 10.6 | 12.5 | 12.3 |
| Other^b^ | 7.9 | 8.0 | 8.7 | 8.8 | 7.9 | 8.1 | 6.8 | 6.7 |
| **US Region** |  |  |  |  |  |  |  |  |
| Northeast | 19.3 | 19.3 | 21.9 | 21.6 | 19.8 | 19.8 | 15.1 | 15.0 |
| Midwest | 24.5 | 24.2 | 24.6 | 24.3 | 23.1 | 23.4 | 24.4 | 24.1 |
| South | 38.5 | 38.6 | 35.8 | 36.0 | 40.1 | 39.5 | 43.0 | 43.2 |
| West or other region | 17.6 | 18.0 | 17.7 | 18.1 | 17.0 | 17.4 | 17.5 | 17.8 |
| **Low-income subsidy** | 40.2 | 36.2 | 34.7 | 30.7 | 39.9 | 37.4 | 46.0 | 43.3 |
| **Tobacco or alcohol use** | 7.7 | 6.7 | 4.8 | 4.2 | 7.6 | 6.9 | 10.8 | 9.6 |
| **Drug use disorder** | 2.1 | 1.8 | 0.9 | 0.7 | 2.1 | 2.1 | 3.3 | 3.1 |
| **Index pain diagnosis^c^** |  |  |  |  |  |  |  |  |
| Musculoskeletal | 85.9 | 86.9 | 86.5 | 87.6 | 20.1 | 20.3 | 85.3 | 86.5 |
| Neuropathic | 15.8 | 14.7 | 15.2 | 14.0 | 100.0 | 100.0 | 16.4 | 15.1 |
| Idiopathic | 2.1 | 2.1 | 0.8 | 0.9 | 0.7 | 0.7 | 3.6 | 3.6 |
| **Comorbidities affecting pain treatment** | |  |  |  |  |  |  |  |
| Cardiovascular disease | 73.8 | 70.8 | 71.3 | 67.7 | 75.5 | 72.6 | 76.4 | 73.6 |
| Pulmonary condition | 45.8 | 42.8 | 40.6 | 38.0 | 46.5 | 42.9 | 52.0 | 48.4 |
| Diabetes | 36.9 | 35.6 | 34.8 | 33.5 | 50.0 | 49.7 | 39.0 | 37.9 |
| Mental disorder | 23.2 | 21.1 | 18.4 | 16.3 | 24.2 | 21.7 | 28.1 | 26.0 |
| Gastrointestinal tract disorder | 17.1 | 16.3 | 14.3 | 13.8 | 17.4 | 16.3 | 19.4 | 18.6 |
| Urinary tract infection | 11.9 | 12.2 | 10.1 | 10.4 | 11.7 | 12.1 | 13.3 | 13.4 |
| Kidney disease | 14.4 | 13.3 | 12.7 | 11.4 | 16.4 | 15.0 | 16.3 | 15.1 |
| Fall or fracture | 8.7 | 8.1 | 5.7 | 5.3 | 6.4 | 6.1 | 10.4 | 9.6 |
| Neurodegenerative disease | 6.0 | 5.8 | 5.1 | 4.9 | 7.0 | 6.8 | 6.8 | 6.4 |
| Liver disease | 5.1 | 4.7 | 4.2 | 3.8 | 5.5 | 5.0 | 5.8 | 5.5 |
| **Health care utilization** |  |  |  |  |  |  |  |  |
| Any hospitalization stay | 12.2 | 11.6 | 8.5 | 8.1 | 10.9 | 10.4 | 14.7 | 13.8 |
| Any ED visit | 17.7 | 16.9 | 12.3 | 11.7 | 17.3 | 16.6 | 21.6 | 20.9 |
| Any hospital surgical procedure | 4.5 | 4.1 | 2.2 | 2.0 | 3.3 | 3.2 | 5.6 | 5.1 |

Abbreviations: ADRD, Alzheimer disease and related dementias; ED, emergency department.

^a^Defined as the 6 months prior to the date of a randomly selected chronic pain diagnosis for each patient.

^b^Included Hispanic, Asian, Pacific Islander, and Native American individuals.

^c^Measured as primary or secondary diagnosis as the index diagnosis

**eTable 5. Demographic and Clinical Characteristics of Residents at Nursing Homes Who Have Chronic Pain With or Without ADRD Using Inverse Propensity Weighting, by Cohort**

| Baseline characteristic^a^ | Patients with chronic pain, % | | Opioid-naive patients, % | | Patients with neuropathic pain, % | | Patients with prescription opioids, % | | Patients with moderate to severe pain, %^b^ | |
| --- | --- | --- | --- | --- | --- | --- | --- | --- | --- | --- |
|  | **With ADRD** | **Without ADRD** | **With ADRD** | **Without ADRD** | **With ADRD** | **Without ADRD** | **With ADRD** | **Without ADRD** | **With ADRD** | **Without ADRD** |
| **Total No.** | 37,242 | 4,898 | 23,287 | 2,594 | 2,222 | 548 | 17,618 | 3,130 | 11,029 | 2,203 |
| **Age**, y |  |  |  |  |  |  |  |  |  |  |
| Mean (SD) | 81.1 (11.1) | 80.6 (11.3) | 81.7 (11.0) | 81.3 (11.4) | 75.8 (11.6) | 74.3 (11.5) | 80.3 (11.2) | 79.8 (11.2) | 79.5 (11.3) | 78.9 (11.4) |
| 50-64 | 9.9 | 10.2 | 9.2 | 9.9 | 19.4 | 20.4 | 10.9 | 10.7 | 12.2 | 12.6 |
| 65-74 | 16.2 | 16.3 | 15.0 | 15.2 | 25.7 | 28.0 | 17.8 | 17.7 | 19.2 | 19.6 |
| 75-84 | 28.7 | 28.0 | 28.5 | 26.8 | 28.7 | 27.8 | 29.0 | 29.3 | 29.3 | 27.8 |
| ≥85 | 45.2 | 45.5 | 47.3 | 48.2 | 26.2 | 23.7 | 42.3 | 42.3 | 39.4 | 40.1 |
| **Female** | 74.0 | 73.2 | 71.7 | 70.5 | 69.1 | 71.5 | 76.5 | 75.5 | 77.0 | 77.0 |
| **Race/ethnicity** |  |  |  |  |  |  |  |  |  |  |
| White | 80.4 | 81.6 | 78.8 | 81.1 | 78.3 | 77.2 | 82.9 | 83.8 | 84.5 | 84.1 |
| Black | 14.0 | 13.0 | 15.0 | 13.8 | 14.8 | 15.0 | 12.7 | 11.5 | 11.5 | 11.7 |
| Other^c^ | 5.5 | 5.3 | 6.2 | 5.0 | 6.9 | 7.8 | 4.4 | 4.6 | 4.0 | 4.2 |
| **Region** |  |  |  |  |  |  |  |  |  |  |
| Northeast | 22.7 | 21.5 | 27.0 | 25.7 | 22.2 | 20.1 | 17.1 | 16.0 | 19.1 | 18.5 |
| Midwest | 27.3 | 28.7 | 26.3 | 28.6 | 27.1 | 29.8 | 28.7 | 30.1 | 30.8 | 32.3 |
| South | 39.1 | 39.3 | 36.3 | 36.5 | 37.5 | 36.1 | 42.8 | 42.7 | 39.2 | 38.5 |
| West or other region | 10.9 | 10.5 | 10.4 | 9.2 | 13.2 | 14.0 | 11.4 | 11.2 | 10.9 | 10.8 |
| **Low-income subsidy** | 82.1 | 80.8 | 80.6 | 78.7 | 84.3 | 84.3 | 82.2 | 81.5 | 83.6 | 83.2 |
| **Tobacco or alcohol use** | 5.8 | 6.5 | 5.0 | 5.2 | 7.1 | 6.9 | 7.1 | 6.9 | 7.8 | 7.3 |
| **Drug use disorders** | 1.9 | 2.0 | 1.2 | 0.9 | 2.1 | 2.9 | 2.7 | 3.1 | 3.0 | 3.6 |
| **Index pain diagnosis**^d^ |  |  |  |  |  |  |  |  |  |  |
| Musculoskeletal | 91.6 | 91.7 | 92.9 | 92.9 | 12.3 | 13.1 | 89.8 | 90.0 | 89.4 | 89.5 |
| Neuropathic | 6.8 | 6.9 | 6.4 | 6.7 | 100.0 | 100.0 | 7.7 | 7.3 | 8.0 | 8.2 |
| Idiopathic | 3.1 | 3.1 | 1.8 | 1.6 | 0.6 | 0.4 | 4.7 | 4.9 | 4.8 | 4.8 |
| **Comorbidities affecting pain treatment** |  |  |  |  |  |  |  |  |  |  |
| Cardiovascular disease | 92.7 | 93.0 | 91.4 | 91.0 | 91.8 | 91.1 | 93.9 | 94.6 | 93.9 | 94.1 |
| Pulmonary condition | 57.7 | 59.2 | 53.9 | 53.3 | 58.3 | 54.8 | 62.2 | 62.8 | 64.2 | 64.9 |
| Diabetes | 43.5 | 41.1 | 41.1 | 38.7 | 68.0 | 67.0 | 46.1 | 44.1 | 47.6 | 46.1 |
| Mental disorder | 58.4 | 57.3 | 54.5 | 53.2 | 59.9 | 55.7 | 61.6 | 61.3 | 64.1 | 62.3 |
| Gastrointestinal tract disorder | 43.0 | 43.5 | 40.2 | 39.6 | 44.1 | 44.9 | 45.1 | 46.9 | 45.7 | 47.6 |
| Urinary tract infection | 39.6 | 37.5 | 36.2 | 33.9 | 39.1 | 35.1 | 43.0 | 40.2 | 43.3 | 40.3 |
| Kidney disease | 27.2 | 24.0 | 24.3 | 20.4 | 33.1 | 28.3 | 30.5 | 26.1 | 31.0 | 27.8 |
| Fall or fracture | 23.0 | 25.2 | 18.9 | 22.1 | 17.0 | 12.2 | 27.0 | 30.4 | 26.7 | 28.1 |
| Neurodegenerative disease | 15.7 | 17.6 | 14.8 | 16.6 | 18.8 | 15.5 | 16.3 | 18.6 | 17.2 | 17.9 |
| Liver disease | 5.6 | 5.2 | 4.9 | 4.1 | 6.9 | 6.4 | 6.5 | 5.5 | 6.9 | 6.1 |
| **Health care utilization** |  |  |  |  |  |  |  |  |  |  |
| Any hospitalization stay | 31.6 | 30.5 | 28.8 | 28.2 | 32.6 | 29.1 | 35.5 | 34.4 | 37.1 | 34.5 |
| Any ED visit | 26.4 | 27.9 | 21.9 | 22.6 | 26.1 | 25.7 | 30.6 | 32.6 | 30.9 | 30.8 |
| Any hospital surgical procedure | 7.9 | 7.3 | 6.3 | 5.6 | 8.2 | 7.0 | 10.2 | 9.4 | 10.4 | 10.4 |
| **ADL dependence**^d^ |  |  |  |  |  |  |  |  |  |  |
| None | 23.8 | 27.4 | 23.4 | 27.2 | 24.1 | 27.7 | 24.0 | 28.1 | 27.1 | 29.1 |
| Mild | 31.1 | 31.3 | 31.3 | 31.2 | 27.5 | 25.9 | 31.2 | 29.8 | 31.7 | 31.9 |
| Moderate | 29.1 | 28.0 | 28.7 | 27.2 | 31.1 | 30.5 | 29.8 | 29.0 | 28.0 | 27.4 |
| Severe | 16.0 | 13.4 | 16.6 | 14.4 | 17.2 | 15.9 | 15.0 | 13.1 | 13.1 | 11.6 |
| **PHQ-9 depression symptoms**^d^ |  |  |  |  |  |  |  |  |  |  |
| None | 78.7 | 79.0 | 80.3 | 82.1 | 79.8 | 81.2 | 76.4 | 75.7 | 74.4 | 72.0 |
| Mild | 14.7 | 15.6 | 13.8 | 13.6 | 14.3 | 14.6 | 16.3 | 17.7 | 17.4 | 20.4 |
| Moderate | 4.9 | 4.0 | 4.6 | 3.3 | 4.3 | 3.4 | 5.3 | 4.8 | 5.9 | 5.6 |
| Severe | 1.6 | 1.4 | 1.3 | 1.0 | 1.6 | 0.9 | 2.0 | 1.8 | 2.2 | 2.0 |
| **BMI**^d^ |  |  |  |  |  |  |  |  |  |  |
| Underweight | 7.8 | 6.6 | 7.8 | 6.7 | 5.5 | 4.7 | 8.0 | 6.4 | 7.2 | 6.7 |
| Normal weight | 34.9 | 34.9 | 36.6 | 38.6 | 26.0 | 25.0 | 32.6 | 33.2 | 29.9 | 28.8 |
| Overweight | 28.9 | 28.9 | 30.0 | 28.0 | 29.2 | 30.0 | 27.3 | 27.4 | 26.8 | 28.3 |
| Obese | 28.4 | 29.6 | 25.6 | 26.7 | 39.3 | 40.2 | 32.1 | 33.0 | 36.1 | 36.3 |
| **Nursing home stay duration,** mean (SD), **d^e^** | 318 (76.2) | 311 (66.3) | 318 (75.9) | 311 (66.2) | 302 (90.4) | 292 (78.9) | 312 (81.9) | 306 (71.9) | 319 (76.5) | 312 (64.8) |

Abbreviations: ADRD, Alzheimer’s disease and related dementias; ADL, activities of daily living; BMI, body mass index (calculated as weight in kilograms divided by height in meters squared); ED, emergency department; MDS 3.0, Minimum Data Set, version 3.0; PHQ-9, patient health questionnaire-9

^a^Defined as the 6 months prior to the date of a randomly selected chronic pain diagnosis for each patient.

^b^Defined as having ≥1 quarterly MDS 3.0 pain assessment with a numeric rating scale score of ≥4 or moderate or severe pain based on the verbal descriptor scale.

^c^Included Hispanic, Asian, Pacific Islander, and Native American individuals.

^d^ Measured as primary or secondary diagnosis as the index diagnosis

^e^Measured based on the first observe quarterly MDS 3.0 assessment during 12 months after diagnosis of chronic pain, which was randomly selected per patient.

^f^Measured during the entire 12 months after diagnosis of chronic pain, which was randomly selected per patient.

**eFigure 1. Flowchart of Included Patients**

1,696,991 adults aged ≥50 y with primary or secondary diagnosis of chronic pain from 2011 to 2015

938,845 excluded due to less than 18 months of continuous Medicare enrollment, including 6 months before (baseline) and 12 months (follow-up) after a randomly selected chronic pain (index diagnosis)

758,146 older adults with at least one 18-month continuous enrollment period

57 excluded due to missing MDS 3.0 information on Patient Health Questionnaire-9 or activities of daily living

- 17,623 excluded due to insurance coverage from Health Maintenance Organizations or employers during the 18-month period
- 187,093 excluded due to cancer, hospice care, or palliative care during the 18-month period

13,300 with moderate to severe pain in follow-up

20,750 with prescription opioids in follow-up

2,820 with neuropathic pain as index diagnosis

25,966 naive to opioids in baseline

42,245 with chronic noncancer

pain

225, 687 with prescription opioids in follow-up

74,467 with neuropathic pain as index diagnosis

352,191 naive to opioids in baseline

511,128 with chronic noncancer pain

511,128 resided in community

(75,258 with ADRD and 435,870 without ADRD)

42,245 resided in nursing homes

(37,117 with ADRD and 5,128 without ADRD)

553,430 older adults with chronic noncancer pain

**eFigure 2.** Absolute Standardized Differences for Baseline Demographic and Clinical Characteristics of Community-Dwelling Older Patients Who Had Chronic Pain With or Without ADRD in the Original Population and After Inverse Probability Weighting


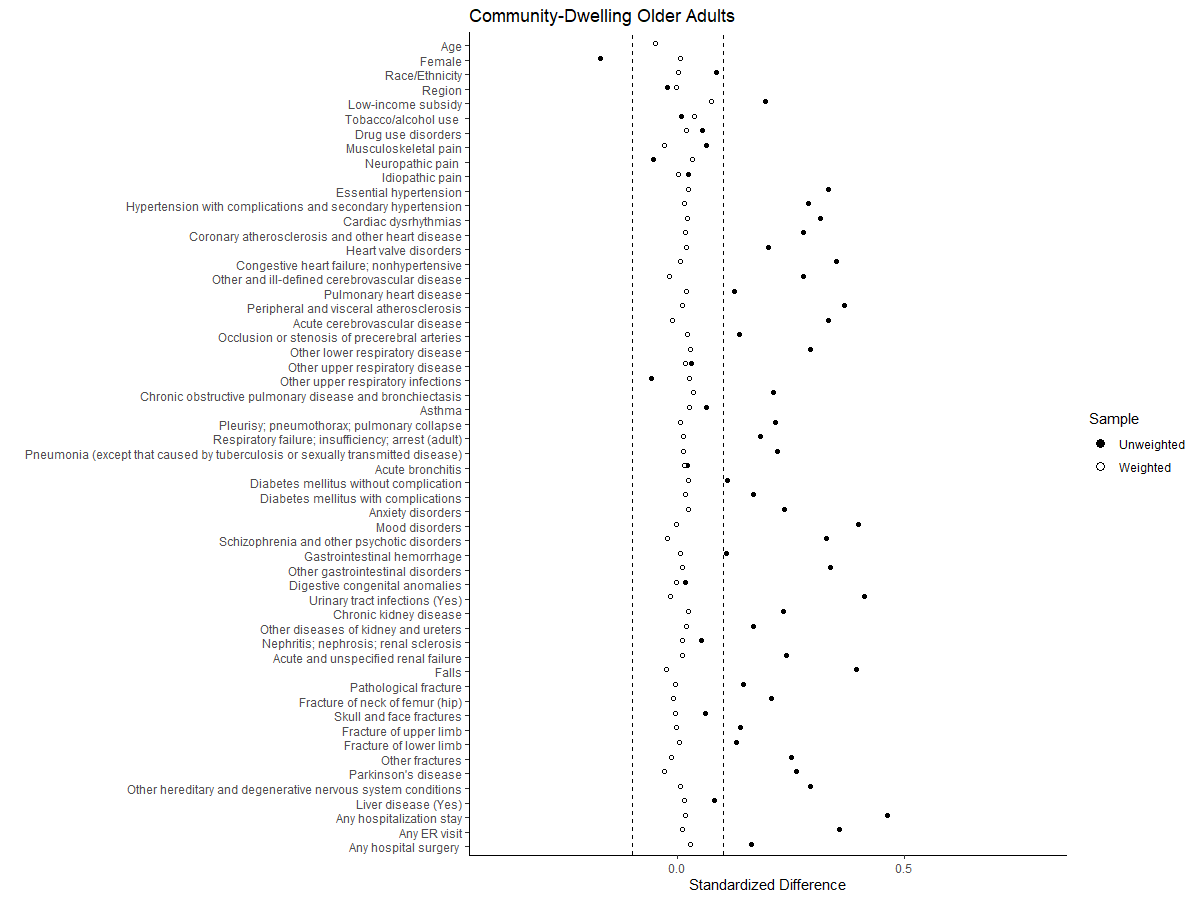


**efigure 3**. Absolute Standardized Differences for Baseline Demographic and Clinical Characteristics of Nursing Home Residents Who Had

Chronic Pain With or Without ADRD in the Original Population and After Inverse Probability Weighting


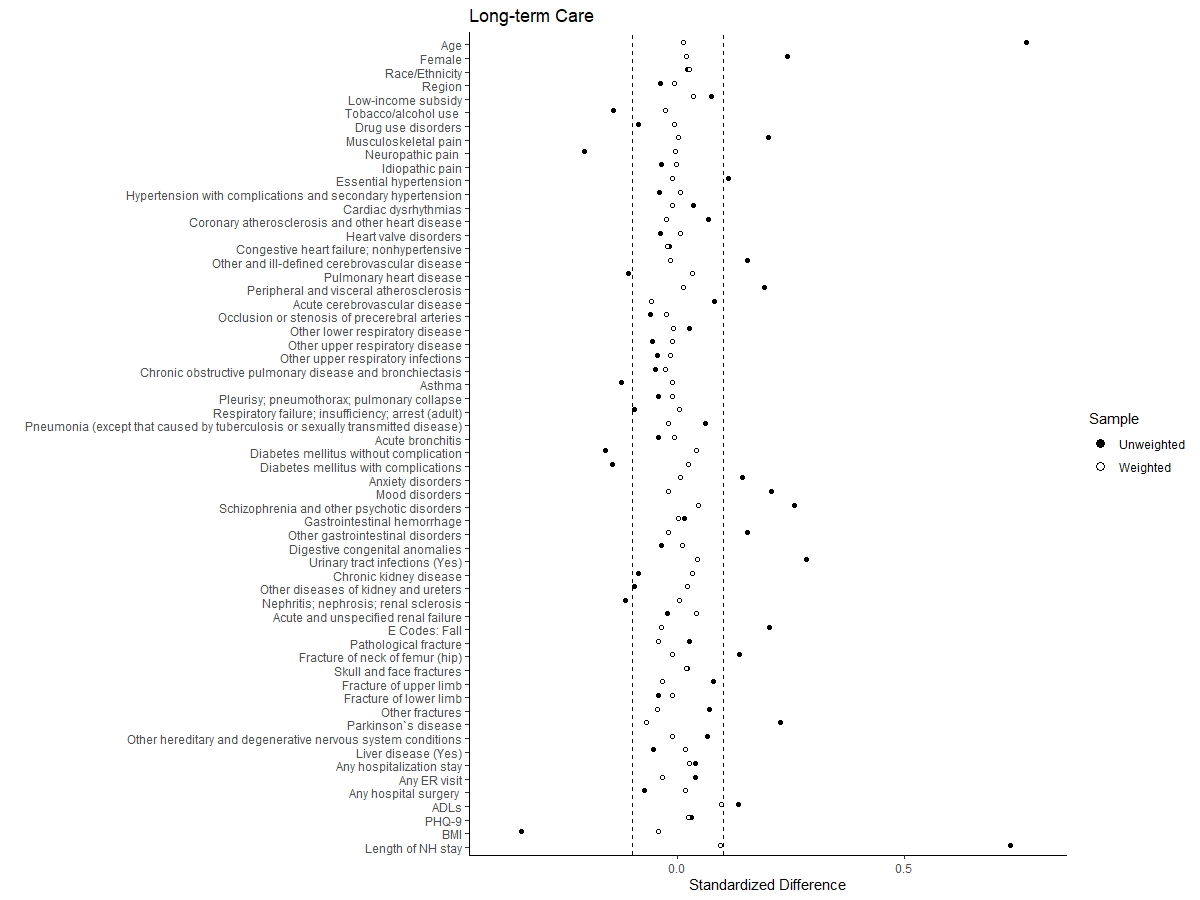

Supplement: Supplementary file 1 — Additional file 1: Table S1. ICD-9-CM Codes and Procedures for Disease Conditions and Service Care Considered in the Study. Table S2. Medications of Interest in This Study. Table S3. Current and Recent Guideline and Consensus Documents on Opioid Prescribing for Older Adults with Noncancer pain. Table S4. Characteristics of Community-Dwelling Patients with Chronic Pain With or Without ADRD Using Inverse Propensity Weighting, by Cohort. Table S5. Characteristics of Nursing Home Residents Who Have Chronic Pain With or Without ADRD Using Inverse Propensity Weighting, by Cohort. Figure S1. Flowchart of Included Patients. Figure S2. Absolute Standardized Differences for Baseline Demographic and Clinical Characteristics of Community-Dwelling Patients With Chronic Pain With or Without ADRD in the Original Population and After Inverse Probability Weighting. Figure S3. Absolute Standardized Differences for Baseline Demographic and Clinical Characteristics of Nursing Home Residents Who Had Chronic Pain With or Without ADRD in the Original Population and After Inverse Probability Weighting. [file 13195_2021_818_MOESM1_ESM.docx]
